# The Presentation and Management of Facial Artery Pseudoaneurysm: A Review of the Literature

**DOI:** 10.4274/tao.2020.5594

**Published:** 2021-03-26

**Authors:** Suparna Roy, Neha Jain

**Affiliations:** 1 Department of Otorhinolaryngology, Chacha Nehru Bal Chikitsalaya Hospital, Delhi, India

**Keywords:** Facial artery, pseudoaneurysm, post traumatic, arteriography, false aneurysm, infant

## Abstract

Pseudoaneurysm is a rare vascular complication of trauma, causing an incomplete tear of the vessel wall. We present a clinical case report arising from the distal branch of facial artery in an infant. Facial artery pseudoaneurysm is a rare complication of facial trauma and can easily be misdiagnosed especially in the paediatric age group. Prompt investigation and diagnosis with timely and apt intervention is the key to the successful management of facial artery pseudoaneurysm.

## Introduction

Pseudoaneurysm is a rare vascular complication of trauma, causing an incomplete tear of the vessel wall (1). The common presentation is a painless pulsatile swelling usually associated with a palpable thrill and an audible bruit (2). The preferred imaging modalities to clinch the diagnosis of such lesions are computed tomography (CT), arteriography, and ultrasonography (USG). Treatment options include compression, surgical resection, ligation of the involved vessel without resection, selective arteriography with embolization and intralesional sclerotherapy (3).

We present a case of facial artery pseudoaneurysm along with its clinical, radiological findings and management. To our knowledge, this is the first reported surgically managed case of distal facial artery pseudoaneurysm in an infant.

## Case Presentation

**Figure 1:**
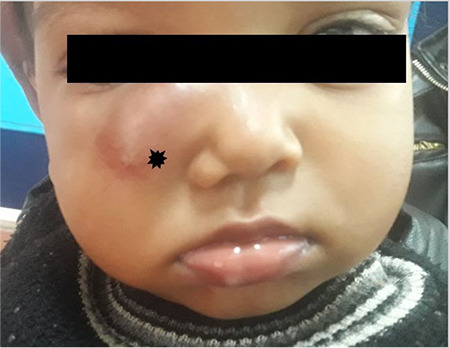
Right cheek swelling 3.0x2.5 cm (multi-point star)

A one-year-old girl presented with a right cheek mass that had been growing for three weeks. She had fallen from her bed one month ago. Clinical examination revealed a 3x2.5 cm, oval, ill-defined, reddish-purplish colored mass involving the right cheek and extending from the medial canthus to the right malar region, causing facial disfigurement (Figure 1). On palpation the mass was tense, firm, non-tender and pulsatile. Keeping differentials of A-V malformation and pseudoaneurysm in mind, color Doppler USG and CT scan were performed.

**Figure 2:**
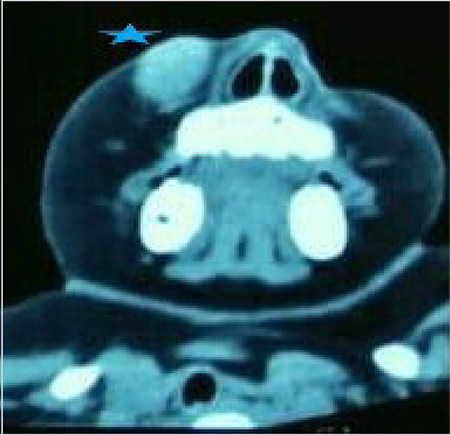
Contrast enhanced CT imaging showed an enhancing well-defined ovoid mass (5-point star) on the right side of the face with no bony erosion CT: Computed tomography

**Figure 3:**
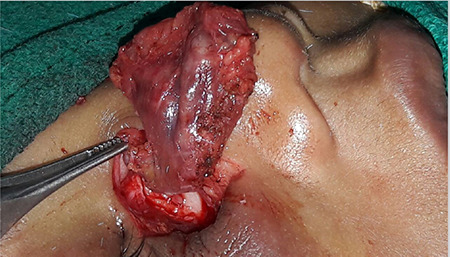
Vascular mass arising from distal branch of right facial artery was excised in toto

**Figure 4:**
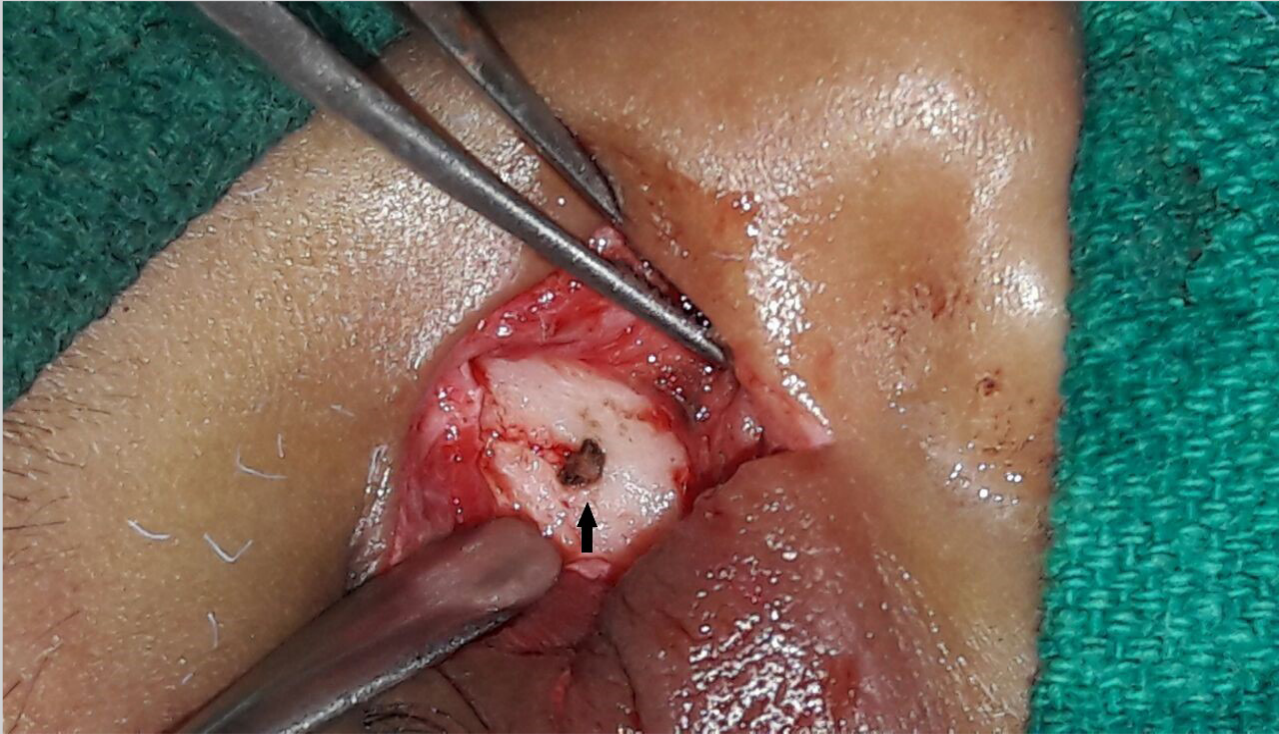
Distal branch of facial artery was ligated and secured (black arrow)

Color Doppler USG revealed a well-defined vascular cystic lesion in the right cheek highly suggestive of a pseudoaneurysm. CT images were suggestive of a large vascular mass; however, the exact origin of the lesion could not be confirmed (Figure 2).  The child was taken up for surgical excision under general anaesthesia. The mass was dissected in toto and was found to be arising from the distal branch of the facial artery (Figures 3, 4). A standard pressure dressing was applied for 48 hours. The postoperative period was uneventful, and the child recovered well. Histopathological examination confirmed the diagnosis of pseudoaneurysm.

## Discussion

Aneurysms are classified as true, false, or dissecting. True aneurysm is a dilation of all the three layers of the intact vessel wall (1). False aneurysm or pseudoaneurysm occurs when blood leaks through an injured blood vessel into the surrounding tissues with a persistent communication or connection between them (1, 2).

Incomplete tear of the involved vessel wall causes blood to flow into the surrounding tissue resulting in tamponade and clot formation (1). Hemorrhage persists until the pressure in the periarterial zone equals the mean arterial pressure (4). Ultimately, the hematoma organizes. The perivascular connective tissue forms an endothelial lined sac leading up to the pseudointima (4). Eventually, the hematoma liquefies. The result is a communication between the artery and the aneurysmal sac, forming a pulsating mass. The persistent arterial pressure results in gradual expansion of the false aneurysm. The final outcome is further growth or rupture of the pseudoaneurysm. Thus, the time between the trauma and the clinical presentation of the pseudoaneurysm varies from days to years (1).

Facial artery pseudoaneurysms are rare because of the small diameter of the facial artery and its deep and protected location (4). Trauma, exposure to radiation, infection, undernutrition and malignancy are the risk factors for the formation of a pseudoaneurysm (1, 4). The classical presentation is a painful pulsatile swelling usually associated with a palpable thrill, an audible bruit or unexplained neurological deficit (1). On rare occasions, they present as a non-pulsatile mass due to thrombin formation or deep-seated location (5). Sometimes, pseudoaneurysm may rupture and cause hemorrhage (1).

The diagnostic tool of choice is arteriography (2). Arteriography outlines the feeding vessels and localizes the exact anatomic site of bleeding (6). Differentials to be kept in mind include lipoma, cyst, simple hematoma, abscess, A-V fistula, inflamed lymph node and neuroma (7). The final diagnosis of pseudoaneurysm is made by histopathological examination (1).

Non-invasive treatment modalities include compression and observation, but this modality is time consuming (1). Invasive treatment modalities include surgical resection, ligation of the feeding vessel, selective arteriography with embolization and percutaneous injection (3).

Classically, treatment of pseudoaneurysm has always been open surgical exploration with vessel ligation, but with the recent advancements in minimally invasive surgery, endovascular approach has become more popular. Endovascular management entails treatment aimed only at the aneurysm or the entire vessel from which the pseudoaneurysm arises (8). 

Percutaneous embolization is performed by direct injection of thrombin under ultrasound guidance. This transforms the pseudoaneurysm into hematoma, which then resorbs in due course of time (9). Using the Seldinger technique, diagnostic arteriogram and therapeutic embolization can be performed in the same setting. This procedure is more selective with minimal risk of neural injury and scarring (10). For embolization, the different materials used are micro-coils, gel foam, polyvinyl alcohol particles, n-butyl cyanoacrylate glue, detachable balloons (2).

In the present case, mass was the result of a blunt trauma. Considering the superficial location of the mass, and the limited facilities for endovascular surgery in our center, surgical excision and ligation of the feeding vessels were planned. Outcome was good and no recurrence was reported in the 18-month follow-up period. The involvement of the distal facial artery makes this case all the more interesting and unique.

We recommend keeping pseudoaneurysm as a differential diagnosis in palpable pulsatile swellings of the head and neck. And an algorithm based on site (proximal/distal/superficial/deep), presentation, availability of facilities and clinical expertise should be formulated and considered for management of this uncommon entity. Although arteriography with embolization is the preferred method, surgical resection is also a safe and effective method for the treatment of head and neck pseudoaneurysms. An open procedure can be considered in cases of superficial swellings, failure of endovascular approach, or as in our case, non-availability of endovascular surgery resources.

## Conclusion

Facial artery pseudoaneurysm is a rare complication of facial trauma. Prompt investigation and diagnosis with timely and apt intervention is the key to the successful management of facial artery pseudoaneurysm. Surgical resection is a safe and effective treatment method and can be considered in cases of superficial swellings, failure of endovascular approach, or as in our case, non-availability of endovascular surgery resources.
